# Robust structural analysis of native biological macromolecules from multi-crystal anomalous diffraction data

**DOI:** 10.1107/S0907444913001479

**Published:** 2013-06-13

**Authors:** Qun Liu, Qinglian Liu, Wayne A. Hendrickson

**Affiliations:** a New York Structural Biology Center, NSLS X4, Building 725, Brookhaven National Laboratory, Upton, NY 11973, USA; b Department of Physiology and Biophysics, Virginia Commonwealth University, Richmond, VA 23298, USA; c Howard Hughes Medical Institute, Columbia University, New York, NY 10032, USA; d Biochemistry and Molecular Biophysics, Columbia University, New York, NY 10032, USA; e Department of Physiology and Cellular Biophysics, Columbia University, New York, NY 10032, USA

**Keywords:** anomalous scattering, multiple crystals, phase determination, sulfur SAD

## Abstract

Structure determinations for biological macromolecules that have no known structural antecedents typically involve the incorporation of heavier atoms than those found natively in biological molecules. Currently, selenomethionyl proteins analyzed using single- or multi-wavelength anomalous diffraction (SAD or MAD) data predominate for such *de novo* analyses. Naturally occurring metal ions such as zinc or iron often suffice in MAD or SAD experiments, and sulfur SAD has been an option since it was first demonstrated using crambin 30 years ago; however, SAD analyses of structures containing only light atoms (*Z*_max_ ≤ 20) have not been common. Here, robust procedures for enhancing the signal to noise in measurements of anomalous diffraction by combining data collected from several crystals at a lower than usual X-ray energy are described. This multi-crystal native SAD method was applied in five structure determinations, using between five and 13 crystals to determine substructures of between four and 52 anomalous scatterers (*Z* ≤ 20) and then the full structures ranging from 127 to 1200 ordered residues per asymmetric unit at resolutions from 2.3 to 2.8 Å. Tests were devised to assure that all of the crystals used were statistically equivalent. Elemental identities for Ca, Cl, S, P and Mg were proven by *f*′′ scattering-factor refinements. The procedures are robust, indicating that truly routine structure determination of typical native macromolecules is realised. Synchrotron beamlines that are optimized for low-energy X-ray diffraction measurements will facilitate such direct structural analysis.

## Abstract 2

Anomalous diffraction signals from typical native macromolecules are very weak, frustrating their use in structure determination. Here, native SAD procedures are described for enhancing the signal to noise in anomalous diffraction by using multiple crystals are described. Five applications demonstrate that truly routine structure determination is possible without the need for heavy atoms.

## 1. Introduction   

The generation of images of macromolecules from X-ray diffraction of crystals requires the retrieval of phases, which are lost in the recording of X-ray diffraction patterns. When structures of sufficient similarity are already known for molecular relatives, the method of molecular replacement very often provides adequate initial approximations for full structural analysis. Otherwise, phases must be evaluated *de novo* either by direct methods or by experimental phase evaluation. Until now, the dominant means of *de novo* phase evaluation has entailed derivatization with heavy atoms such as Hg (*Z* = 80) for phase evaluation by multiple or single isomorphous replacement (MIR or SIR) or by multi- or single-­wavelength anomalous diffraction (MAD or SAD) (Hendrickson, 1991 ▶, 1999 ▶). Reliable incorporation of selenomethionine (*Z* = 34) into proteins (Hendrickson *et al.*, 1990 ▶) provides particularly efficient *de novo* structure determination by Se MAD/SAD phasing. For proteins that naturally contain metals, such as Fe (*Z* = 26), structure determination by native SAD or MAD is often straightforward. Typical anomalous diffraction signals are only a few percent of the normal diffraction signals, and experimental noise from various sources (counting statistics, diffuse scattering, absorption, radiation damage *etc.*) can obscure the detection of the anomalous differences needed to place the dozens of atomic positions in a typical substructure and then to obtain phases for full structure analysis.

Complications from experimental noise can adversely affect even SAD/MAD analyses of selenomethionyl (SeMet) proteins, and they can be devastating for the feebler anomalous signals from lighter atoms such as sulfur (*Z* = 16). The problems are exacerbated for crystals that diffract poorly owing to high atomic mobilities. These difficulties are evident in the struggles with SeMet phasing at relatively low resolution and with sulfur SAD phasing even at medium resolution. Such struggles are mostly known only by anecdote, but the effects are evident in the holdings of the Protein Data Bank. As of this writing, more than 5200 *de novo* SAD structures have been reported in the Protein Data Bank; however, structures determined at relatively higher resolution and with stronger anomalous scatterers (*Z* > 20) predominate at the 98% level. We can identify only 32 SAD structures at low resolution (*d*_min_ ≥ 3.5 Å) and we find only 58 SAD structures based on light-atom (*Z* ≤ 20) anomalous scatterers (Table 1 ▶). This underrepresentation of low-resolution and low-*Z* SAD structures poses a challenge for crystallographic methods.

Biological macromolecules generically contain low-*Z* elements that are potentially adequate for effective native SAD experiments: sulfur in proteins and phosphorus in nucleic acids. Proteins on average have methionine and cysteine residues at frequencies of 1.42 and 0.68%, respectively, corresponding to one S atom every 30 residues. Nucleic acids have one P atom per base. Routinely effective native SAD analysis could greatly reduce the uncertainties and complications that arise from heavy-atom derivatization and incorporation. The concept of native SAD phasing was first demonstrated three decades ago by the resolved anomalous phasing of crambin (Hendrickson & Teeter, 1981 ▶). SAD as we now know it emerged as density-modification procedures (Wang, 1985 ▶; Cowtan & Main, 1993 ▶) became effective for breaking the phase ambiguity intrinsic to single-source phase evaluation, as in an application to neurophysin (Chen *et al.*, 1991 ▶). Its extension to sulfur-SAD phasing evolved with feasibility tests on lysozyme (Dauter *et al.*, 1999 ▶) and the *de novo* analysis of obelin (Liu *et al.*, 2000 ▶). Substantial advances have been made during the past 30 years; among these are the inverse-beam mode of data collection (Hendrickson *et al.*, 1989 ▶), the use of highly redundant data collection (Dauter & Adamiak, 2001 ▶; Weiss *et al.*, 2001 ▶), the use of longer wavelengths (Yang *et al.*, 2003 ▶), the use of special crystal mounts (Kitago *et al.*, 2010 ▶; Sugahara, 2012 ▶), the use of a fine-slicing low-noise detector (Mueller *et al.*, 2012 ▶) and other developments (as reviewed in Dodson, 2003 ▶; Dauter, 2006*a* ▶). Nevertheless, as shown in Table 1 ▶, the output of light-atom-only native SAD structures has been low and largely restricted to crystals that diffract very well. It appears that difficulties in making accurate measurements of the weak anomalous signals from native biological macromolecules adversely affect the outcomes.

In attempting to enhance the signal-to-noise ratio for anomalous diffraction experiments, one can contemplate increasing the anomalous signal strength and also reducing the noise. For resonant-edge experiments, such as those used for SeMet proteins, anomalous signals might be strengthened through enhanced energy resolution. For light-atom experiments, as for sulfur SAD, the signals might be enhanced through the increase in the *f*′′ scattering strength at lower X-­ray energy. In either case, noise that obscures weak signals might be reduced through the averaging of diffraction data. To increase the multiplicity within the constraints of radiation damage, we have adopted the approach of measuring data from several statistically equivalent crystals. In previously addressing difficult phasing problems at low resolution, we were able to solve a relatively large and poorly diffracting SeMet protein structure by merging data sets from eight crystals at 3.5 Å resolution (Liu, Zhang *et al.*, 2011 ▶). We showed that various measures of anomalous signal accuracy were improved substantially with multi-crystal data and that averaged data robustly supported both substructure determination and phasing.

Here, we have devised robust procedures for multi-crystal native SAD experiments at lower than usual X-ray energy. We used these procedures to solve five protein structures varying in size (127–1200 ordered residues) and the number of anomalous scatterers (4–52) at modest resolutions (2.3–2.8 Å). The five applications are diverse. HK9_S_ is an example that could not be labeled with SeMet owing to nonexpression or expression in inclusion bodies. Netrin G2 is a human protein and various heavy-atom derivatizations did not yield satisfactory electron-density maps. CysZ is a novel membrane transporter. TorT–TorS_S_ is a heteromeric protein–protein complex at a relatively low resolution of 2.8 Å. DnaK–ATP is the largest *de novo* native SAD structure, with 1200 ordered residues in the asymmetric unit. The structure determinations are summarized in Table 2 ▶. Results from the analysis of four of them have recently been published separately (Liu *et al.*, 2012 ▶). Here, we use the application to DnaK–ATP to illustrate the process of multi-crystal native SAD phasing. We also use the combined experience from the five applications to characterize features of solvability, radiation damage and identification of light-element ions (Na^+^, Mg^+^, Cl^−^, K^+^ and Ca^2+^) from the refinement of *f*′′ scattering factors. We conclude that robust structure analysis of native biological macromolecules is feasible when SAD data from multiple statistically equivalent crystals are combined appropriately.

## 2. Materials and methods   

Details of the production of proteins and crystals for the HK9_S_, netrin G2, CysZ and TorT–TorS_S_ analyses are described in the supplementary material of our previous paper (Liu *et al.*, 2012 ▶) and in the associated structure reports (Brasch *et al.*, 2011 ▶; Moore & Hendrickson, 2012 ▶; Qi *et al.*, 2013 ▶). Many of the procedures used for data collection, data reduction and structural analysis have also been described previously. Here, we use the specific application to DnaK in complex with ATP (DnaK–ATP) in order to demonstrate the procedures that are employed.

### 2.1. Protein production, crystallization and data collection   

For this study, we used a nearly full-length construct from the bacterial Hsp70 chaperone DnaK from *Escherichia coli* with two mutations. Detailed protein-construct description and protein production will be published separately (Qi *et al.*, 2013 ▶). Briefly, a DnaK construct encoding residues 1–610 was affinity-purified and concentrated to 10–­20 mg ml^−1^ in a buffer consisting of 5 m*M* HEPES pH 7.5, 10 m*M* KCl and then brought to 5 m*M* magnesium acetate, 2 m*M* ATP just before being set up for crystallization. DnaK–ATP crystals were grown at 277 K by the hanging-drop vapor-diffusion method. An equal volume of protein solution was mixed with well solution consisting of 1.8–2.0 *M* ammonium sulfate, 0.1 *M* HEPES pH 7.5, 2–3% PEG 400. For cryocrystallography, the crystals were briefly soaked in well solution supplemented with 15% glycerol and were then flash-cooled in liquid nitrogen for data collection at 100 K. Standard Hampton loops were used for crystal mounting and, since the crystals were relatively large, no special precautions were taken to remove cryo-solution around the crystals.

X-ray diffraction data were collected from the five best-diffracting crystals using an ADSC Q4R CCD detector on beamline X4A at the National Synchrotron Light Source (NSLS). The X-ray wavelength was tuned to the Fe *K* edge (λ = 1.743 Å) as verified by fluorescence scans. The inverse-beam mode of data collection was used; the crystals were rotated 180° every ten frames to measure Friedel mates. Each frame had a rotation angle of 0.5°. A total of 400 × 2 frames were collected from each of the five crystals, with an exposure time of 12 s per frame.

### 2.2. Diffraction data reduction and analyses   

Single-crystal data sets were processed using the *XDS* (Kabsch, 2010*a* ▶,*b* ▶) and *CCP*4 (Winn *et al.*, 2011 ▶) programs *POINTLESS* and *SCALA* (Evans, 2011 ▶). Frames for a single crystal were indexed and integrated by *XDS*. The same crystal orientation matrix was used for both sides of the inverse-beam sweeps. Integrated intensities were then corrected by *XDS* for detector modulation, radiation damage and absorption. Bijvoet pairs were treated differently during all corrections and were kept separate after *XDS*. The *CCP*4 programs *POINTLESS* and *SCALA* were then used for further data combination, scaling and merging. Ambiguities in multi-crystal data sets owing to random indexing among crystals were resolved by re-indexing in *POINTLESS* before combining. Reduction of single-crystal as well as multi-crystal data was carried out using *SCALA*. During data reduction, Bijvoet pairs were treated as equivalent reflections during scaling but were treated separately during merging. Rotational scale and *B*-factor restraints were used with corrections for secondary beam and absorption. After processing of single-crystal data sets, unit-cell parameters and diffraction intensities were used for outlier crystal detection and rejection by clustering analyses (Liu *et al.*, 2012 ▶). Compatible data sets were then scaled and merged together by *SCALA*. Relative anomalous correlation coefficients (RACCs) of individual data sets to the merged data set were calculated for further outlier crystal rejection. For RACC calculations, data were truncated at 3.5 Å resolution. Data sets with RACC values below 35% were rejected from further analyses. After outlier crystal rejection, the remaining data sets were reordered according to their RACC values and were progressively merged either from the best to the worst or from the worst to the best for structure-determination attempts. The program *CTRUNCATE* (French & Wilson, 1978 ▶; Padilla & Yeates, 2003 ▶) was used to convert merged intensities to structural amplitudes with Bijvoet pairs separated. Data-collection and reduction statistics for single-crystal and multiple-crystal data sets are listed in Table 3 ▶.

### 2.3. Phasing   

Anomalous center substructures were found by *SHELXD* (Sheldrick, 2010 ▶). *SHELXD* first derives *E*-values from Bijvoet-difference amplitudes, |Δ*F*_±*h*_| = ||*F*(*h*)| − |*F*(−*h*)||, and excludes the weak *E*_obs_ data, defined by *E*_min_, from the substructure searches as being ineffective and unreliable. The program uses correlation coefficients (CCs) between *E*_obs_ and *E*_calc_ as criteria to evaluate the validity of substructure solutions. CC_all_ is the correlation coefficient based on all data and CC_weak_ is that from the 30% of the weak data which, akin to *R*_free_, is not used in substructure searches and provides a sensitive measure of validity (Schneider & Sheldrick, 2002 ▶). For *SHELXD* searches, we tried various high-resolution cutoffs from 2.5 to 5 Å in steps of 0.1 Å. Based on these attempts, we used a high-resolution cutoff at 3.8 Å for substructure determination with an *E*_min_ value of 1.6 to search for 32 protein sulfur sites by assuming the presence of two DnaK–ATP molecules in the crystallographic asymmetric unit. Attempts were also made using single-crystal data sets as well as their merged data. For each data set, 1000 or 10 000 attempts were made.

The substructures found by *SHELXD* were refined and completed using *Phaser* (Read & McCoy, 2011 ▶). The log-likelihood gradient maps produced by *Phaser* were used for substructure completion. This is implemented in *Phaser* by the LLG complete keywords ‘COMPLETE ON’ and ‘SIGMA 5.5’. For phasing experiments, initial SAD phases to the data limit were calculated by *Phaser*. Phases for both enantiomorphs were generated and these phases were then subjected to automatic density modification with solvent flattening and histogram matching as implemented in the *CCP*4 program *DM* (Cowtan & Zhang, 1999 ▶). Although the crystallographic asymmetric unit contained two DnaK–ATP molecules related by twofold noncrystallographic symmetry (NCS), such NCS was not used for density modification. The estimated solvent content of 68% was used for solvent flattening and no additional treatments were made. After density modifications, the resulting electron-density maps were visually checked to identify the correct hand by looking for more interpretable molecular boundaries and structural features.

For those single-crystal and multi-crystal data sets that did not support successful substructure determinations by *SHELXD*, the substructures obtained from the merged five-crystal data were used for SAD phasing. Similarly, SAD phases to the data limit were calculated by *Phaser* and initial phases were density-modified for further analyses.

### 2.4. Structure refinement and analyses   

Initial automatic model building was carried out using *ARP*/*wARP* (Langer *et al.*, 2008 ▶) starting with experimental electron densities after density modification. Manual model building was carried out using *Coot* (Emsley *et al.*, 2010 ▶) and refinements were performed using *phenix.refine* (Adams *et al.*, 2011 ▶; Afonine *et al.*, 2012 ▶). TLS parameters and isotropic *B* factors were refined without NCS restraints. Friedel pairs were treated as two reflections in refinement. The refined model was validated by *PROCHECK* (Laskowski *et al.*, 1993 ▶) and *MolProbity* (Chen *et al.*, 2010 ▶) to ensure good geometry. To identify anomalous scatterers, Bijvoet-difference Fourier maps were visually inspected. The *f*′′ values of respective anomalous scatters were then refined against the merged data. The *f*′′ refinements were performed by *phenix.refine* with starting values of zero for all anomalous scatterers.

## 3. Results   

### 3.1. Experimental considerations   

**Figure 1:**
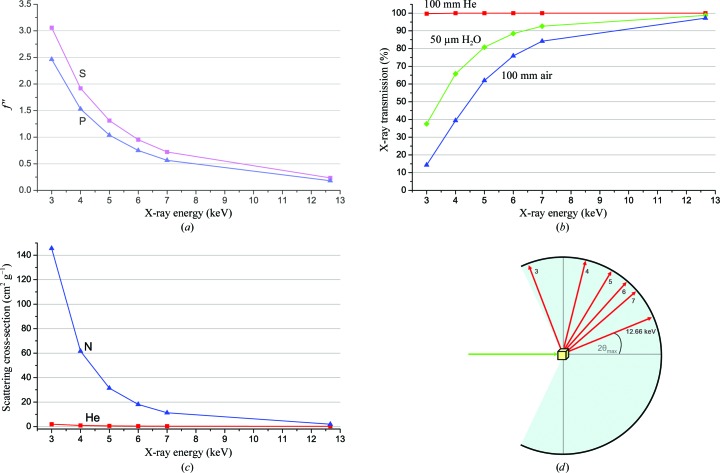
Experimental considerations of low-energy experiments. (*a*) Theoretical anomalous signals from sulfur (magenta) and phosphorus (blue) (*f*′′, in electrons) as a function of X-ray energy. (*b*) X-ray transmission through 100 mm beam paths of air (blue) and helium (red) or through a 50 µm thickness of water (green) as a function of energy. (*c*) Scattering cross-section for nitrogen (blue) and helium (red) as a function of X-ray energy. (*d*) Requirement of detector geometry for low-energy experiments in order to cover Bragg spacings to 2.5 Å.

For native SAD experiments, there are various considerations for optimized anomalous signal measurement. Perhaps the most critical parameter of concern is the X-ray energy. Although anomalous signals from light elements increase steadily with decreasing energy, many other properties affect diffraction experiments increasingly adversely with decreasing energy. The advantages of low energy are readily apparent: *f*′′ for sulfur increases from 0.24 e at the Se *K* edge (12.7 keV) to 3.05 e at 3 keV, and the profile for phosphorus is similar (Fig. 1 ▶ *a*). Factors that militate against this advantage include X-ray absorption, diffuse scattering and large Bragg angles. Absorption of X-rays by air, as in the path of diffracted rays, or by water, as in the crystal sample, diminishes the diffracted rays (Fig. 1 ▶ *b*), incoherent scattering into the background increasingly obscures Bragg diffraction as absorption increases at lower energies (Fig. 1 ▶ *c*) and, by Bragg’s law, lower energy diffraction patterns extend to greater diffraction angles, making for challenging diffraction geometries (Fig. 1 ▶ *d*).

**Figure 2:**
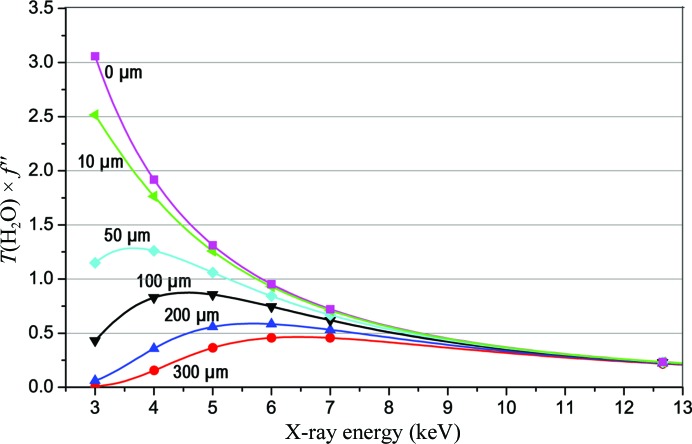
Dependence of transmitted anomalous signals on X-ray energy. Water is taken to approximate the absorptivity of typical macromolecular crystals, and we plot the product of the anomalous signal from sulfur (*f*′′, in electrons) with the X-ray transmission through various thicknesses of water (red, 300 µm; blue, 200 µm; black, 100 µm; cyan, 50 µm; green, 10 µm; magenta, 0 µm) as a function of X-ray energy. Symbols identify the Se *K* edge energy and selected relevant low-energy values.

There are solutions for many of the complicating low-energy factors. Importantly, both absorption and scattering in the beam paths can be mitigated by replacing air with an atmosphere of helium (Figs. 1 ▶ *b* and 1 ▶ *c*). With respect to detector geometry, we have already shown that it is possible to record a full 3.2 Å resolution data set at 3.7 keV (U *M*_IV_ edge) on a flat detector (Liu *et al.*, 2001 ▶); however, the preferred solution is to use a shaped (geodesic dome or cylindrical) detector. On the question of absorption in the diffracting sample, it obviously helps to use mounts that present the crystal cleanly (Kitago *et al.*, 2010 ▶; Sugahara, 2012 ▶); however, the crystal itself is an unavoidable source of absorption, which of course depends on the crystal thickness. Approximating X-­ray absorption by macromolecular crystals as that of water (macromolecular crystals are 30–80% water and the absorptivity of proteins and nucleic acids is not far off that of water), we can characterize the trade-off between crystal size and anomalous scattering signal by plotting the transmitted signal as a function of sample size (Fig. 2 ▶). Although other factors, including incoherent scattering, absorption in optical elements and windows, detector efficiency and radiation damage, will contribute to energy optimization, crystal size is an important determinant. As such, the optimum for a 200 µm sample is at ∼6 keV, whereas that for a 50 µm sample is at ∼4 keV. To aspire to lower energy and larger transmitted anomalous signals, true microcrystals and microbeams are needed for optimization.

The crystals used for the four initial tests of multi-crystal SAD phasing were in the size range 100–300 µm and we chose to set the energy to 7.112 keV (Fe *K* edge calibrated by iron-­foil fluorescence). The crystals of DnaK–ATP were all 200–300 µm in thickness, where the predicted optimum is in the 6–­7 keV range, so we also used the Fe *K* edge in DnaK–ATP experiments. The inverse-beam mode of data collection (Hendrickson *et al.*, 1989 ▶; Clemons *et al.*, 2001 ▶) was used, but a helium cone was not used since the DnaK–ATP crystals diffracted very strongly.

### 3.2. Criteria for outlier crystal rejection   

When combining diffraction data from multiple crystals, it is important to assure that the data sets to be merged are statistically compatible with one another. We did not find any incompatible data sets in our previous eight-crystal SeMet SAD phasing (Liu, Zhang *et al.*, 2011 ▶), which was at relatively low resolution (*d*_min_ ≥ 3.5 Å); however, the current native SAD studies are based on weaker signals and are at higher resolutions. Thus, more stringent standards for crystal uniformity might be needed. We thus devised three tests for statistical equivalence and applied them for outlier rejection. The results of these tests for our four initial applications of multi-crystal SAD phasing have been described previously (Liu *et al.*, 2012 ▶). Here, we show the comparisons for the DnaK–ATP crystals. Except for one CysZ crystal, all of the other crystals used in these five structure analyses are statistically compatible and contribute collectively.

**Figure 3:**
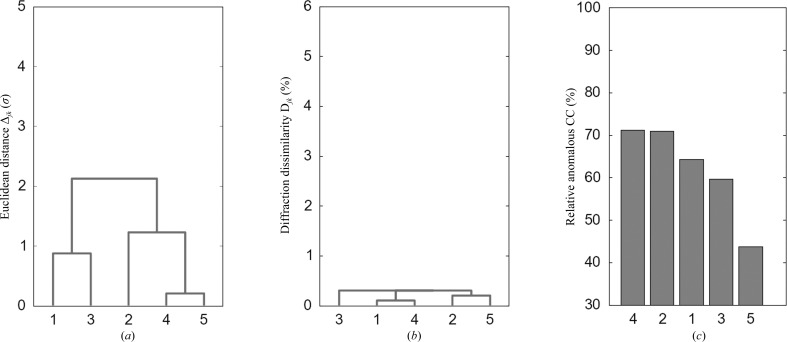
Outlier crystal rejection. Variations among crystals from multi-crystal data sets. (*a*) Cluster analyses of unit-cell variations. (*b*) Cluster analyses of overall diffraction dissimilarity. (*c*) Relative anomalous correlation coefficient. Unit-cell variations are standard Euclidean distances normalized by population variances, *i.e.* the distance between *j* and *k* among *N* crystals is Δ_*j*,*k*_ = 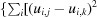/*V*_*i*_]}^1/2^, where *u_i_* includes all variable unit-cell parameters *i*, each having a variance of *V*_*i*_ = σ_*i*_^2^ = 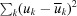/*N*, *k* = 1→*N*. The overall diffraction dissimilarity between crystals *j* and *k* is defined as *D*_*i*,*j*_ = 1.0 − C_*i*,*j*_, where *C*_*i*,*j*_ is the correlation coefficient between all Bragg intensities in common between the two diffraction patterns.

Our first criterion for crystal compatibility is a unit-cell deviation analysis. In the particular case of DnaK–ATP crystals, which belong to space group *I*422, we first used the two variable unit-cell parameters *a* and *c* to calculate Euclidean distances between pairs (*j*, *k*) of crystals. These distances were then used to construct a distance matrix, normalized by population variances, which was used for a clustering analysis for the five crystals (Fig. 3 ▶ *a*). Crystals 1 and 3 form one cluster and crystals 2, 4 and 5 form a second cluster, which appeared to be compatible for averaging at an inter-cluster distance just over 2σ. When a large number of crystals are available, structural analyses for individual clusters may be informative.

Our second criterion was a further check by diffraction dissimilarity analysis. We define diffraction dissimilarity as 1 − PICC, where the paired intensity correlation coefficient (PICC) is the correlation coefficient between intensities in a pair of diffraction patterns. Bijvoet pairs were averaged for PICC calculations, and for DnaK–ATP the high-angle data were truncated to 3.5 Å resolution. The 1 − PICC distances were used directly for clustering analysis by searching for the maximum distances between two clusters. From the diffraction dissimilarity clustering (Fig. 3 ▶ *b*) the five crystals differ very little, all within 0.5%, indicating that these five crystals are compatible.

Our third criterion is a test on anomalous diffraction signals. Anomalous differences from our native SAD experiments proved to be too small for useful pairwise analysis, so we devised a relative anomalous correlation coefficient (RACC) whereby the merged Bijvoet differences |Δ*F*_±*h*_| in the data set from an individual crystal are compared with those in the merged data set averaged from all crystals. For DnaK–ATP, high-angle data were truncated to 3.5 Å resolution for RACC calculations and the results are shown with crystals ordered by RACC rank (Fig. 3 ▶ *c*). The top two crystals have RACC values over 70% and the lowest still shows a significant contribution at 44%, again implying that all five crystals are compatible with one another. To optimize structure-determination efficiency, we reordered the crystals on the basis of their RACC values (Table 3 ▶).

Although each criterion may have its limitations and could falsely reject or retain crystals, we find that in general, as here for DnaK–ATP, these criteria are consistent with one another. Based on experience from these five native SAD structures, we suggest that a unit variation of <3σ, a diffraction dissimilarity of <5% and an RACC of >35% are acceptable values for including crystals. Meeting all three criteria reinforces the assurance that all merged crystals are statistically equivalent. While it seems advantageous to reject data from outlier crystals, the process is robust even to the inclusion of outliers. As long as the number of crystals is large, a few outlier crystals are unlikely to affect overall SAD phasing results.

### 3.3. Diffraction signal strength   

In order to assess the strength of diffraction signals during the course of our native SAD phasing analyses, we followed three statistical measures: the anomalous correlation coefficient (ACC), which compares two randomly selected subsets from a data set, the average Bijvoet difference 〈|Δ*F*|〉 normalized by the normalized standard deviation σ(〈|Δ*F*|〉) of that averaged difference, which we denote simply as Δ*F*/σ(Δ*F*), and the average diffracted intensity 〈*I*〉 normalized by the standard deviation σ(〈*I*〉) of that averaged difference, which we denote simply as *I*/σ(*I*). Results from the progressions in adding of crystals in our first four applications of native SAD phasing have already been reported (Liu *et al.*, 2012 ▶), and here we describe results from progressions in the DnaK–ATP analysis.

**Figure 4:**
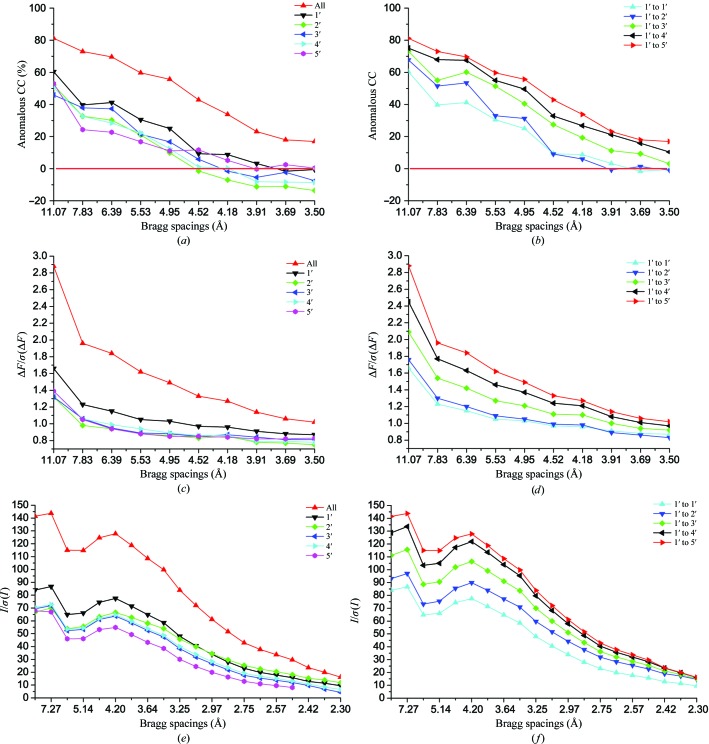
Diffraction signal strength in single-crystal and multi-crystal data. (*a*), (*c*), (*e*) Anomalous correlation coefficients (anomalous CC), Δ*F*/σ(Δ*F*) and *I*/σ(*I*) for single-crystal data and merged data as a function of scattering-vector length, |*S*| = 2sinθ/λ, which is labeled as the Bragg spacing, *d* = 1/|*S*|. (*b*), (*d*), (*f*) Diffraction signal strength by progressive mergings of the single-crystal data shown in (*a*), (*c*) and (*e*) together. All single-crystal data sets were reordered based on relative anomalous correlation coefficients. Data sets are identified by the inset keys.

ACC values were used as reported by the *CCP*4 program *SCALA* and they are strongly resolution-dependent. For the single-crystal data sets, ACC values approach 45–60% at low scattering angles and then fall below 10% for Bragg spacings greater than 4.5 Å (Fig. 4 ▶ *a*); however, the ACC values were substantially enhanced across all Bragg spacings upon merging of the five single data sets, with progressive enhancement as data were added crystal by crystal from the best to the worst (Fig. 4 ▶ *b*). Even the worst crystal 5′ helped to increase the ACC. Overall, the ACC increased from 14.2% for the best single crystal 1′ to 41.8% for the merged data (Table 3 ▶).

Profiles of Δ*F*/σ(Δ*F*) with respect to Bragg spacings also demonstrated enhancements of anomalous signals from the inclusion of multiple crystals. As for ACC, there is a marked improvement in anomalous signal-to-noise ratios upon merging (Fig. 4 ▶ *c*) and this is again progressive with crystal-by-crystal mergings (Fig. 4 ▶ *d*). The best single-crystal data set, from crystal 1′, is appreciably stronger than all others; yet nevertheless the merged data set shows strongly enhanced signal to noise even relative to this best single set. Overall, Δ*F*/σ(Δ*F*) for the merged data to 3.5 Å spacings was enhanced by a factor of 1.52 over the 1′ data set (Table 3 ▶). Moreover, even the worst single-crystal data set, from crystal 5′, still boosts *ΔF*/σ(Δ*F*) by 12% over the 1′–4′ merging.

*I*/σ(*I*) values provide measures of signal to noise in overall diffraction and they thus define the limit of measureable diffraction. This limit is at 2.3 Å Bragg spacing for DnaK–ATP (except for crystal 5, which was truncated at 2.5 Å). As for ACC and Δ*F*/σ(Δ*F*), the *I*/σ(*I*) profiles also increased dramatically for merged over individual data sets (Fig. 4 ▶ *e*) and progressively upon addition crystal by crystal (Fig. 4 ▶ *f*). The notable difference is the extension to higher resolution for overall diffraction compared with the much weaker anomalous diffraction signals.

### 3.4. Substructure determination   

The first step in native SAD phasing is to determine the substructure of anomalous scatterers. For these studies, we have used the combined Patterson search and *Shake-and-Bake* dual-space refinement of *SHELXD* (Sheldrick, 2010 ▶). Details of substructure determinations for the first four native SAD phasing studies have been reported previously (Liu *et al.*, 2012 ▶); thus, we concentrate here on the DnaK–ATP studies to illustrate the procedures. As in the other applications, optimized *SHELXD* parameters feature a resolution cutoff substantially reduced from the diffraction cutoff: to 3.8 from 2.3 Å for DnaK–ATP.

Compared with other cases, the DnaK–ATP crystals proved to be quite recalcitrant to substructure determination. We first performed tests with 1000 attempts for each data set in the crystal-by-crystal progression and found no solutions until data from all five crystals were merged. Subsequently, we in­creased the number of tries to 10 000 per data set. We found no solutions for any of the single-crystal data sets even at this level; however, we did then find solutions for the four-crystal cases both in the best-to-worst order (1′ to 4′) and in the reverse order (5′ to 2′), as well as the five-crystal case (Tables 4 ▶ *a* and 4 ▶ *b*). In all cases, the vast majority of attempts resulted in candidate solutions characterized by correlation coefficients CC_all_ and CC_weak_ clustered at 15 ± 1% and 2 ± 1%, respectively. True solutions have correlation coefficients that are significantly higher than this random background; in the four-crystal 1′ to 4′ case, 20 successful solutions emerged with CC_all_/CC_weak_ values of up to 37.2/22.4% and adding the fifth crystal further improved the success rate and CC values (Table 4 ▶ *a*).

As an aside, it is worth noting that a single successful *SHELXD* solution suffices for substructure determination. Each of the successful candidates can produce a correct substructure, but identifying even one is a challenge when the success rate is low. The success rates for DnaK–ATP (0.4% for the five-crystal case) were significantly worse than in the other multi-crystal native SAD experiments (from 0.7% for TorT–TorS_S_ to 35% for HK9_S_; Liu *et al.*, 2012 ▶). The success rate is a function both of the signal-to-noise ratio, which is improved by the inclusion of additional crystals, and of substructure complexity, which is an intrinsic complication. Based on the protein composition and assuming two DnaK molecules per asymmetric unit, 32 S atoms were expected; however, the actual refined substructure included 52 atoms of varying strength (Table 2 ▶). Lower success rates can be expected as the substructure size increases.

**Figure 5:**
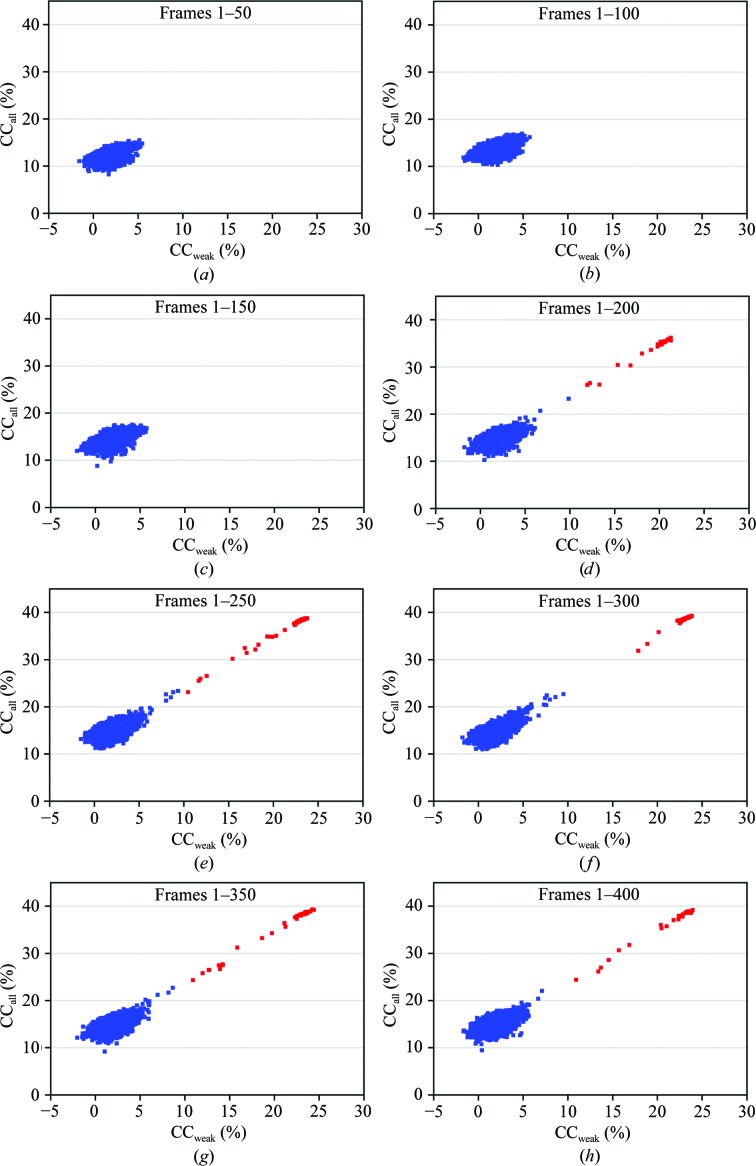
Profiles of *SHELXD* correlation coefficients (CC) between observed and calculated Bijvoet differences. Results are shown from multi-crystal data sets merged as accumulated wedges, as defined in Table 5 ▶(*b*). Each panel shows the distribution of CC_all_ and CC_weak_ values from 10 000 attempts. Successful solutions are colored red and random solutions are colored blue.

To further check the robustness of substructure determination, we merged the data into eight successive wedges (Table 5 ▶ *a*) and used the accumulative wedged data in substructure-determination trials (Table 5 ▶ *b*), using the same parameters as for the crystal-by-crystal trials. Although each 50-frame wedge was at least 99.5% complete, none of the individual wedges supported substructure determination. Furthermore, there were no solutions from accumulative mergers through the first three wedges (frames 1–150); however, successful solutions were obtained from mergers of four or more wedges. In Fig. 5 ▶, we plot the CC_all_ and CC_weak_ distributions from accumulative wedged data sets wedge 1 to wedges 1–8, singling out the successful solutions. Comparing Tables 4 ▶(*a*), 4 ▶(*b*) and 5 ▶(*b*), it can be seen that with accumulative wedged data an overall multiplicity of 74.1 can suffice for substructure determination, whereas with crystal-by-crystal mergers a higher overall multiplicity of 123.3 seems to be needed. We presume that the improved performance from wedged data merging is a consequence of the reduced level of radiation damage in this approach.

### 3.5. Phasing   

Phase determination can proceed once a substructure is known, whether or not the particular data set supported substructure determination. The substructures found from various conditions are all essentially the same except for shifts of the origin and enantiomorphic changes. Therefore, the substructure from the five-crystal data set was used in each subsequent DnaK–ATP phasing test. Initial SAD phase calculations were performed by *Phaser* and phase improvement was performed by density modification with *DM*. Automatic model building into resulting *DM*-modified electron-density maps was performed with *ARP*/*wARP*. The structure based on the five-crystal data set was refined with *PHENIX*. Phasing effectiveness was evaluated by the figure of merit (FOM), by map correlation coefficients (MapCC) comparing experimental electron-density maps with the map produced from the ultimate refined atomic model and by the fraction of ordered structure that could be built by *ARP*/*wARP*.

SAD phasing tests were performed based on each data set described in Tables 4 ▶(*a*), 4 ▶(*b*), 5 ▶(*a*) and 5 ▶(*b*) using the five-crystal substructure for each. The final electron-density map from the five-crystal data set itself was excellent, giving a MapCC of 85.3% and automated building of 1117 of the 1200 residues (93%) for the DnaK–ATP structure. Even the SAD-phased map without *DM* modification gave a MapCC of 46.6%. We also found that data sets that did not support substructure determination on their own could support overall structure determination when given the substructure of anomalous scatterers. Thus, single data set 1′ gave a MapCC of 73.0% and an 88% autobuilt atomic model. Only data from single-crystal set 5′, the worst crystal, and the single-wedge data sets other than wedge 1 did not support automated structure determination efficiently (40% autobuilt for crystal 5′, 39–47% for wedges 2 through 8). With the addition of data, either crystal by crystal or wedge by wedge, progressive improvements followed in the monitors of phasing effectiveness.

There are complications in measuring the effectiveness of SAD phasing in that the quality of a resulting map is a function of the density-modification protocol as well as the anomalous phasing efficacy. Moreover, automated map interpretability depends on the fitting algorithm as well as the map quality. These complications are evident in the enhancement progressions of our tracking parameters. The overall enhance­ments from the single crystal to five-crystal determinations are 34% for FOM; 29% for MapCC before *DM*; 17% for MapCC after *DM* and 5% for residues built correctly for the RACC-ordered progression (Table 4 ▶ *a*); 62, 91, 90 and 132%, respectively, in the reverse-ordered progression (Table 4 ▶ *b*); and 46, 40, 31 and 5%, respectively, for the wedge-by-wedge progression (Table 5 ▶ *b*). For the RACC-ordered progression, MapCC values after *DM* do not improve after the 1′ to 3′ merger and the number of autobuilt residues is already at 88% after the first crystal, 1′ to 1′. Provided that starting SAD phases are of sufficient quality, density modification can proceed to completion; similarly, automated model-building procedures can proceed to completion by injecting atomic refinement information. From these results, *ARP*/*wARP* succeeds at the 90% level when MapCC is above ∼35% before *DM* (∼70% after *DM*). FOM and MapCC before *DM* are purely dependent on the anomalous scattering, but they underestimate the SAD phasing efficacy since they are affected by phase ambiguity. Perhaps the intrinsic figure of merit from bimodal phase probability distributions (Hendrickson, 1971 ▶) could be used as a better measure. In any case, it is important to stress that the initial maps for 17 of the 26 tests represented in Tables 4 ▶ and 5 ▶ could not have been produced were it not for the substructure, which required a minimum of four crystals. Clearly, substructure determination was the most challenging aspect of native-SAD phasing for DnaK–ATP.

**Figure 6:**
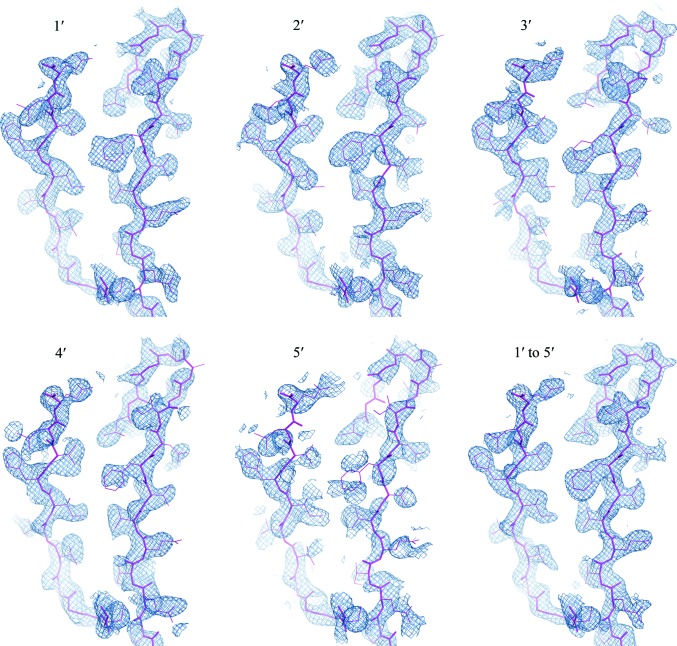
Experimental native SAD electron densities for single as well as merged data sets. Electron-density distributions calculated from phases after density modification at 2.3 Å are shown as sky-blue meshes contoured at 1.5σ. For reference, the model of the refined structure (residues 428–442 and residues 463–480 in molecule *B*) is shown as sticks (magenta). Note the improved continuity and coverage for side chains in merged data 1′ to 5′. This figure was prepared using *PyMOL* (http://www.pymol.org).

**Figure 7:**
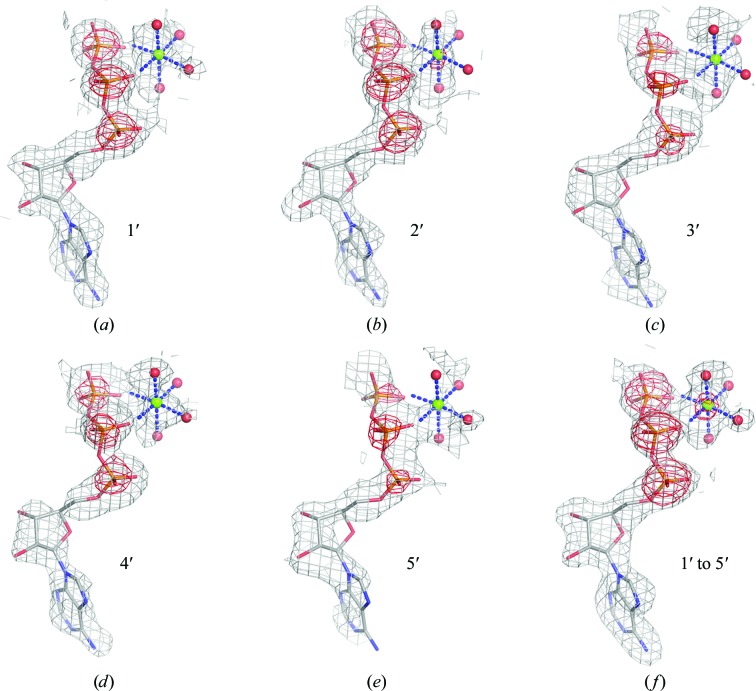
Experimental native SAD and Bijvoet-difference Fourier electron densities for the Mg^2+^-ATP complex. Native SAD distributions, produced as in Fig. 6 ▶, are shown as sky-blue meshes contoured at 1.5σ. Bijvoet-difference Fourier peaks are shown as red meshes contoured at 5σ, showing coincidence with the Mg (*Z* = 12) and P (*Z* = 15) atoms in the refined DnaK–ATP structure.

To visualize the phasing results, we plotted experimental electron densities from the five single-crystal data sets and from the merged five-crystal (1′ to 5′) data set (Fig. 6 ▶). Crystal 1′ shows reasonably continuous electron-density coverage for the main chain and for most side chains (Fig. 6 ▶ *a*), while electron density for the worst crystal, 5′ (Fig. 6 ▶ *e*), would be hard to interpret without being guided by the model. Other crystals showed intermediate density quality. In contrast, the merged five-crystal data set gave strikingly superior electron-density coverage of the model. Similarly, the five-crystal density for the Mg^2+^-ATP complex is better than for any single-crystal case, although even crystal 5′ is interpretable (Fig. 7 ▶). It must be emphasized again that the single-crystal maps depend on assuming a substructure determined from multiple crystals.

### 3.6. Identification of low-­*Z* atoms from native SAD measurements   

Native SAD phasing of proteins can be considered to be sulfur SAD phasing since the S atoms of methionine and cysteine residues are the predominant light atoms in proteins. In our present studies, however, we find that light atoms present in ligands and associated ions are also usually present (Table 2 ▶), and in the case of DnaK–ATP these comprise nearly 40% of the anomalous substructure. The additional sites appear unavoidably from the substructure determination, and their probable identity can often be assigned from the chemical environment. However, independent assurance is also possible. We show here that positive identifications are possible based solely on the anomalous scattering signals at a single X-ray energy remote from the absorption edges.

**Figure 8:**
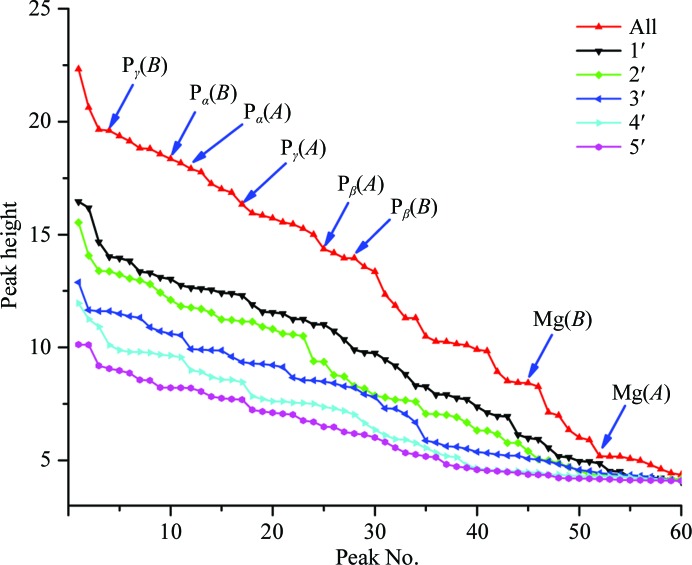
Bijvoet-difference Fourier peak profiles. Ordered peak-height profiles are shown for maps from each single-crystal data set (identified by the inset keys) and for the map from the merged data set (red). Peak heights are given in units of r.m.s.d. over the entire respective Fourier syntheses. Peaks corresponding to the six P atoms and two Mg^2+^ ions of the Mg^2+^-ATP complexes are indicated by blue arrows. Atom entities in molecule *A* or *B* are also indicated in parentheses.

DnaK is an Hsp70 molecular chaperone and its ATPase activity is critical for its function in protein folding (Qi *et al.*, 2013 ▶). For this study, a hydrolysis-deficient mutant protein was crystallized in the presence of 10 m*M* KCl, 5 m*M* magneisum acetate, 2 m*M* ATP and 1.8–2.0 *M* ammonium sulfate. The *SHELXD* analyses returned 45 sites, and the 32 strongest were used for substructure completion and initial SAD phasing by *Phaser* assuming an all-sulfur substructure. After model refinement by *PHENIX*, we generated Bijvoet-difference Fourier maps to cover the two DnaK molecules in the asymmetric unit and used the *CCP*4 program *PEAKMAX* to find the 60 strongest peaks above 3σ (Fig. 8 ▶). By comparing the peak-height profile from the five-crystal data set with the refined atomic model, we matched 32 peaks to protein S atoms, 12 peaks to 11 sulfate ions (one sulfate has two mutually exclusive positions), six peaks to the P atoms of two ATP molecules and two to ATP-associated Mg^2+^ ions. The weakest of these matches, at 5.2σ, is of peak 52 with the Mg^2+^ ion of molecule *A*. This peak coincides with a magnesium ion coordinated octahedrally by four water molecules and by the β and γ phosphate groups of this ATP molecule (Fig. 7 ▶); peak 45, at 8.4σ, corresponds to the Mg atom of molecule *B* (Fig. 8 ▶). Incidentally, the comparison of Bijvoet-difference peak profiles from the five-crystal merger *versus* those from the five single-crystal data sets (Fig. 8 ▶) provides yet another demonstration, beyond those of Fig. 4 ▶, of the power of multi-crystal averaging.

Conventionally, the positive identification of atomic elements from anomalous scattering has entailed measurements above and below an absorption edge or, for lighter elements where measurements at the edge are not readily feasible, the use of heavier same-group replacements such as Br^−^ for Cl^−^, Rb^+^ for Na^+^ or K^+^, and Sr^2+^ or Yb^3+^ for Ca^2+^ or Mg^2+^ with measurements above the surrogate edge. Because of wide differences in atomic mobilities (*B* factors), element identifications from peak heights in Bijvoet-difference Fourier syntheses (Fig. 8 ▶ here and Fig. 2 in Liu *et al.*, 2012 ▶) are fraught, and for ionic species the chemical environment may not be decisive for identification. Thus, we devised a highly effective alternative strategy for these studies. We first carried out refinements of models of the postulated elemental species with Friedel mates averaged; then, again using *phenix.refine* (Afonine *et al.*, 2012 ▶), we followed this with refinements against Friedel-separated data but now including elemental *f*′′ values as variable parameters. Results of scattering-factor refinements for our five native SAD structures are presented in Table 6 ▶. The refinements converged to almost the same values whether started as here with all *f*′′ values set to zero or with initial values of 0.699 e, the theoretical *f*′′ for sulfur at the Fe *K*-edge energy. The process is robust because atomic positions, occupancies and *B* factors are largely determined by the normal scattering components, whereas the fitting of deviations from Friedel’s law requires accurate anomalous scattering factors.

For many of the sites in these five structures, the identity of the anomalous element is not in question. Sulfur is such a case, of course. Thus, by comparing the refined sulfur *f*′′ values with the theoretical value calculated from first principles, one obtains a check of the refinement procedure. The five fitted *f*′′(S_protein_) values average 0.630 ± 0.025 e, which is within 3σ of the theoretical value of 0.699. It is low, however, by a factor of 0.90, which is similar to the average factor by which the other eight entries in Table 6 ▶ are low. Although its origins are unclear, this underestimation does not preclude clear-cut identification of anomalously scattering elements. Notably, the refined *f*′′ value clearly identifies the Mg^2+^ sites in DnaK–ATP, consistent with the Bijvoet-difference map (Fig. 7 ▶). In contrast, previous anomalous diffraction analyses of Hsp70 complexes with Mg^2+^-ADP did not show detectable signals for Mg (Wilbanks & McKay, 1995 ▶; Arakawa *et al.*, 2011 ▶). Moreover, although K^+^ was expected in DnaK–ATP, being relevant for DnaK function and being found bound in complexes of Mg^2+^-ADP with Hsp70 (Wilbanks & McKay, 1995 ▶; Arakawa *et al.*, 2011 ▶) and of Mg^2+^-ATP with Hsp110 (Liu & Hendrickson, 2007 ▶; Polier *et al.*, 2008 ▶), the results from Fig. 8 ▶ and Table 6 ▶ definitively rule out its presence as an ordered component of DnaK–ATP.

### 3.7. Native SAD structures   

**Figure 9:**
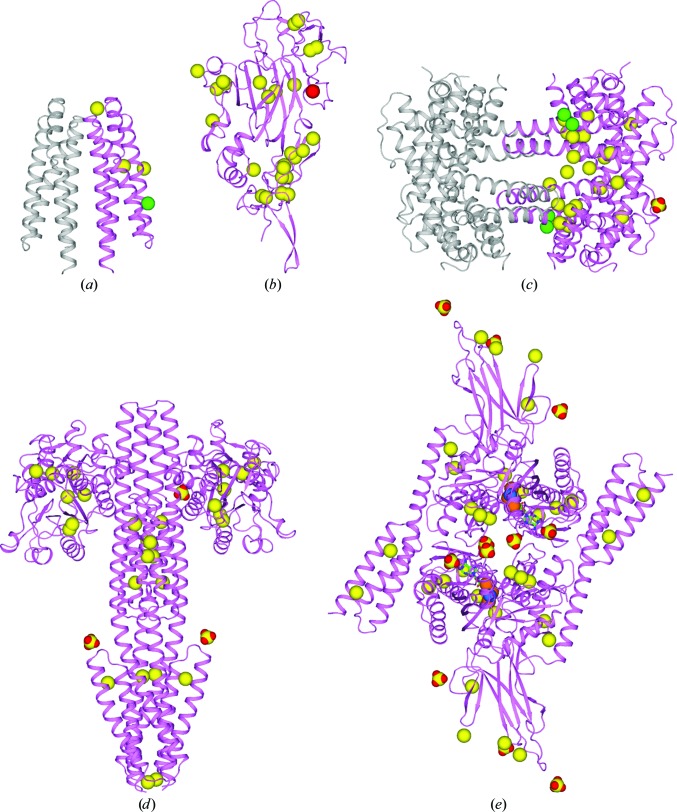
Native SAD structures ordered by the number of residues in the asymmetric unit. (*a*) HK9_S_: a substructure of three S atoms and one Cl atom defined 127 ordered residues at 2.3 Å resolution. (*b*) Netrin G2: a substructure of 26 S atoms and one Ca atom defined 312 ordered residues at 2.3 Å resolution. (*c*) CysZ: a substructure of 20 S atoms, four Cl atoms and one sulfate ion defined 452 ordered residues at 2.3 Å resolution. (*d*) TorT–TorS_S_: a substructure of 28 S atoms and three sulfate ions defined 1148 ordered residues at 2.8 Å resolution. (*e*) DnaK–ATP: a substructure of 32 S atoms, six P atoms, two Mg atoms and 11 sulfate ions defined 1200 ordered residues at 2.3 Å resolution. Each molecular oligomer or complex is shown as a ribbon diagram with those residues in the asymmetric unit colored violet. Anomalously scattering substructures are shown as spheres with S atoms in yellow, Cl atoms in green (HK9_S_ and CysZ), sulfate ions in yellow and red (CysZ, TorT–TorS_S_ and DnaK–ATP) and the one calcium ion in red (netrin G2). The structures shown in (*a*), (*b*), (*c*) and (*d*) were adapted from a previous description (Liu *et al.*, 2012 ▶).

The multi-crystal native SAD procedures described here were devised in the context of applications to five structure determinations (Table 2 ▶), and the resulting structures are shown as ribbon diagrams in Fig. 9 ▶. Structures from three of these analyses, and their associated biological implications, are described in full elsewhere (Brasch *et al.*, 2011 ▶; Moore & Hendrickson, 2012 ▶; Qi *et al.*, 2013 ▶) and descriptions for the other two are in preparation; the initial four structures were also shown in our previous account of this work (Liu *et al.*, 2012 ▶). The five structures range from 127 to 1200 ordered residues, are in crystal lattices from monoclinic to trigonal to tetragonal, have resolution limits from medium at 2.3 Å to low at 2.8 Å and contain systems such as a molecular machine (DnaK), a membrane protein (CysZ) and a protein–protein complex (TorT–TorS_s_). The range of these applications indicates substantial generality and robustness.

## 4. Discussion   

Solving macromolecular structures without needing to incorporate any kind of non-native atom or having a known structural relative is an aspirational idea. Although the proof of concept was demonstrated over 30 years ago (Hendrickson & Teeter, 1981 ▶) and structure determination by SAD was already exploding ten years ago (Table 1 ▶) on the way to its current dominance for *de novo* structure determination, native SAD phasing has not been routinely feasible: witness the fewer than 60 light-atom-only SAD structures in the PDB. Several challenges have thwarted routine *de novo* structure determination and we discuss them here in the context of our approach to native SAD phasing from the merged data of multiple crystals.

### 4.1. Signal-to-noise ratio   

Both because of relatively low signals from light-atom anomalous scatterers and also owing to noise contributions from incoherent scattering at lower X-ray energies, native SAD experiments present challenging signal-to-noise ratios. A protein of 250 residues has approximately eight S atoms on average, which will produce a Bijvoet-diffraction ratio (Δ*F*/*F*) of only ∼1% for data collected at the Fe *K* edge (*E* = 7.112 keV). These are very small signals and they require low noise levels for reliable detection. On average, since σ(Δ*F*) = 2^1/2^σ(*F*), if Δ*F* = *q*|*F*| then Δ*F*/σ(Δ*F*) = (*q*/2^1/2^) × [*F*/σ(*F*)], *I*/σ(*I*) = (1/2) × [*F*/σ(*F*)]; thus, to achieve Δ*F*/σ(Δ*F*) = *a*, one requires *I*/σ(*I*) = (2^1/2^/2) × (*a*/*q*). Thus, at the 1% signal level, to achieve a signal-to-noise level of 1 in average Bijvoet difference, Δ*F*/σ(Δ*F*), requires that *I*/σ(*I*) be at the level of 70. Typical macromolecular diffraction experiments have overall *I*/σ(*I*) values in the range 10–30, well below the required accuracy. Signals can be enhanced by further lowering the X-ray energy (Fig. 1 ▶ *a*); however, the background noise then increases owing to enhanced scattering from samples, mounts, windows and flight-path gases. Such noise can be reduced by experimental design, for example by using a helium beam path (Fig. 1 ▶ *b*), and obfuscating systematic errors can be minimized by strategies such as inverse beam. Noise from random errors can be minimized by increasing the data multiplicity; however, radiation damage limits crystal lifetime and thus single-crystal multiplicity.

The use of multiple crystals seems to be effective for achieving high-multiplicity signal-to-noise enhancement without excessive radiation damage (Liu *et al.*, 2011 ▶). The DnaK–ATP structure determination illustrates the advantages of native SAD experiments well. These crystals have 32 protein S atoms and we find 52 actual anomalous scatterer sites. The calculated Bijvoet-diffraction ratio is close to the value for an average protein: 0.9% and 1.1% at the Fe *K* edge for the respective alternatives, assuming fully occupied sulfur sites; thereby the overall *I*/σ(*I*) of individual data sets ranged between 29.1 (data set 5) and 37.0 (data set 4) (Table 3 ▶), which are all insufficient to provide a signal-to-noise ratio of 1 in Δ*F*/σ(Δ*F*); indeed, none of these data sets supported substructure determination by *SHELXD*. After assuring statistical equivalence among individual data sets (Fig. 3 ▶), we merged them into the five-crystal data set, which has an *I*/σ(*I*) of 64.5 overall and 16.2 in the outermost shell (Table 3 ▶). Bragg spacings for the five-crystal data set still extend beyond 3 Å when truncated at *I*/σ(*I*) > 70, our estimate for signal to noise above 1, and the merged data gave reliable substructure determinations for truncations anywhere between 3.5 and 4.5 Å, where *I*/σ(*I*) values are above 100 (Fig. 4 ▶ *e*).

Another approach that has been proposed for improving accuracy in sulfur anomalous diffraction experiments is a multi-data-set merging procedure (Liu, Chen *et al.*, 2011 ▶). Here, the averaging of *N* data sets exposed at a rate *x*/*N* is shown theor­etically and in practice to improve upon results from a single data set exposed at a rate *x*. From theory (Liu, Chen *et al.*, 2011 ▶), the impact of this multi-data-set procedure is expected to be greatest for intense reflections, which is consistent with an asymptotic *I*/σ(*I*) for intense reflections (Diederichs, 2010 ▶; Liebschner *et al.*, 2012 ▶). We have not performed multi-data-set experiments in this study, but one can expect beneficial complementarity if used in multi-crystal experiments. Nevertheless, for radiation-sensitive samples a single crystal may not suffice, whereas data from multiple crystals can overcome radiation damage and improve phasing efficacy.

### 4.2. Structure solvability   

Native SAD phasing is a two-step process, requiring first determination of the substructure of anomalous scatterers and then the phasing procedure to define the full atomic model. Both steps need accurate anomalous signals and failure in either step normally prevents structure determination, although occasionally one can use initial phases from other sources for substructure determination, as in MR-SAD (Schuermann & Tanner, 2003 ▶; Panjikar *et al.*, 2009 ▶; Read & McCoy, 2011 ▶). Thus, for a typical native SAD structure determination there are two possible bottlenecks. One is the substructure determination, especially if the anomalous substructure is large, and the other is phasing effectiveness if the anomalous substructure is relatively small.

**Figure 10:**
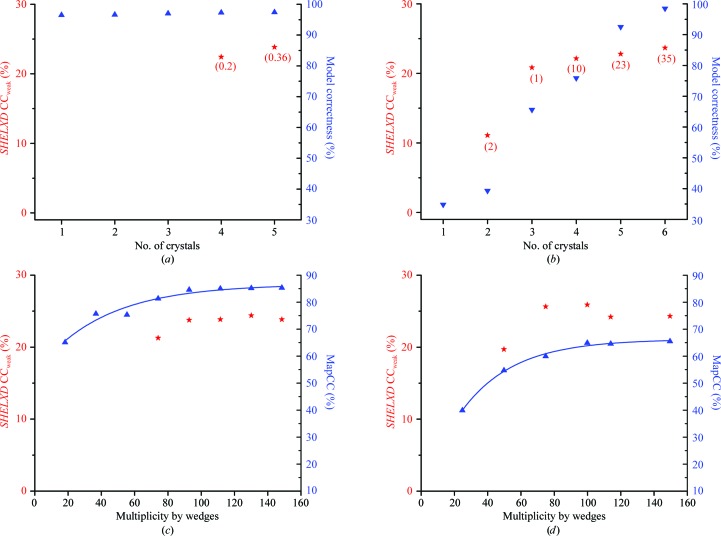
Structure solvability. Parameters of substructure solution (red) and of overall structure determination (blue) are shown in (*a*) and (*b*) for data merged crystal by crystal and in (*c*) and (*d*) for data merged wedge by wedge: (*a*) and (*c*) DnaK, (*b*) and (*d*) HK9_S_. For data merged crystal by crystal, *SHELXD* CC_weak_ values (red stars, success percentages in parentheses) for successful solutions and model correctness (blue triangles) are plotted against number of crystals merged best to worst. For data merged by wedge by wedge, *SHELXD* CC_weak_ and MapCC were plotted with respect to multiplicity in the accumulated wedges. For wedge-by-wedge mergings, each individual data set was divided into wedges of sequentially measured frames (eight wedges for DnaK–ATP and six wedges for HK9_S_) and these data were then merged successively. Successive accumulations from these wedges were then used for native SAD phasing based on substructures obtained previously from analyses of all merged data. MapCC values resulting from each of these successive wedge structures were fitted to an asymptotic formula described previously. Fittings with the asymptotic formula are shown as smooth lines through the data points. Fitted parameters (MapCC_max_, *a*, *b*) are (0.867 ± 0.0190, 0.3791 ± 0.0538, 0.0246 ± 0.0070) for DnaK–ATP and (0.686 ± 0.015, 0.786 ± 0.0788, 0.0258 ± 0.0040) for HK9_S_.

The DnaK substructure has 52 anomalous scatterers, which is relatively high, and substructure determination was the bottleneck in this case. Fig. 10 ▶(*a*) plots the successful *SHELXD* CC_weak_ values and model correctness for structure determination of data merged crystal-by-crystal as in Table 4 ▶(*a*). *SHELXD* could not find a substructure until data were merged from four or five crystals. Once the substructure had been determined, however, it could be used for successful phasing with all other data sets (1′ to 1′, 1′ to 2′, …, 1′ to 5′), resulting in similar model completeness and model correctness (Table 4 ▶ *a* and Fig. 10 ▶ *a*). This again demonstrates that the substructure determination is the rate-limiting factor of multi-crystal native SAD phasing for DnaK and the substructure solvability is four crystals, either when combined best to worst (Table 4 ▶ *a*) or worst to best (Table 4 ▶ *b*).

In contrast to DnaK, the bottleneck for solvability with HK9_S_ was phasing efficacy instead of substructure determination. Fig. 10 ▶(*b*) plots the course of structure determination for HK9_S_ with data merged crystal-by-crystal from best to worst. The four-atom substructure of HK9_S_ could be found by *SHELXD* from as few as two data sets, although three or more data sets gave more reliable substructures and much higher *SHELXD* CC_weak_ values. Moreover, success rates were much higher than for the DnaK analysis: 35% for the six-crystal HK9_S_ experiment compared with 0.36% for the five-crystal DnaK experiment. On the other hand, this substructure could not support successful SAD phasing until five- or six-crystal data were used. The quality of maps improved only gradually with added crystals as measured by automatic building by *ARP*/*wARP* (Fig. 10 ▶ *b*), remaining below 80% model correctness for fewer than five crystals, whereas with six crystals 98 out of 127 ordered residues could be built automatically with a model correctness close to 100%.

These contrasting examples illustrate differing kinds of barriers to structure solution from native SAD data. Although the sticking points for DnaK and HK9_S_ structure determinations differed, it was the signal-to-noise enhancements from multi-crystal averaging that proved to be crucial for both. There is no apparent disadvantage in adding additional crystals to a merged data set; however, the strategy of successive wedge-by-wedge crystal-by-crystal additions until a structure solution appears to be an economical approach.

### 4.3. Radiation damage   

Radiation damage and high multiplicity have conflicting effects in single-crystal data collection: on the one hand, increasing multiplicity can benefit the measurement of weak anomalous signals; on the other hand, radiation damage increases with added X-ray exposure and affects data quality adversely. Radiation damage is a serious problem and is common for delicate samples, for example membrane proteins and large macromolecular complexes.

To detect radiation damage in single-crystal data sets, the *R*_d_ plot (Diederichs, 2006 ▶), anomalous correlation coefficient, scaling *B* factor and *R*_merge_ may all be useful, as have been thoroughly reviewed (Dauter, 2006*b* ▶; Garman & Nave, 2009 ▶; Garman, 2010 ▶; Borek *et al.*, 2010 ▶). For multi-crystal native SAD experiments, these indicators are also effective to detect and reject seriously damaged frames. In addition, the merger of diffraction data by wedges is useful to detect and mitigate the effects of radiation damage (Liu, Zhang *et al.*, 2011 ▶).

Relatively large crystals were available for these studies and complete data sets were collected from each of the crystals in each of the systems. We estimate based on *RADDOSE* simulations (Paithankar *et al.*, 2009 ▶) that accumulated doses were well below the Henderson limit (20 MGy) for all five systems; for DnaK in particular the dose was ∼5 MGy per crystal for the 800 frames in each data set. Nevertheless, our wedge-by-wedge analyses (eight wedges of 50 + 50 inverse-beam wedges each) show clear indications of radiation damage. Although each wedged data set has similar multiplicity and all are essentially complete, the measures of data quality [*I*/σ(*I*), ACC, MapCC before and after *DM*] all worsen on average as exposure increases (Table 5 ▶ *a*). Although *I*/σ(*I*), a measure of overall diffraction quality, deceased from 29.8 for wedges 1–3 to 23.9 for wedges 6–8 (20%), measures sensitive to anomalous signals (ACC and MapCC) suffered more. For example, MapCC after *DM* decreased from 62.2 to 42.3% over the same span (32%).

To test the effects of radiation damage on structure determination, we next merged the DnaK data accumulatively wedge by wedge (Table 5 ▶ *b*) and used these merged data sets for substructure determination and SAD phasing. Measures of substructure determination (by *SHELXD* CC_weak_) and phasing efficacy (by MapCC) were plotted against multiplicity in Fig. 10 ▶(*c*). Similar wedge-by-wedge mergings had been performed before for the other four structures (Liu *et al.*, 2012 ▶) and results from the HK9_S_ study are shown in Fig. 10 ▶(*d*). The features described above with respect to structure solvability in crystal-by-crystal mergings (Figs. 10 ▶ *a* and 10 ▶ *b*) are also observed in wedge-by-wedge mergings (Figs. 10 ▶ *c* and 10 ▶ *d*, respectively); however, performance is better as a function of multiplicity by wedges than of multiplicity measured crystal by crystal, even when in the best-to-worst order. For example, DnaK data merged by accumulated wedges supported substructure determination at an overall multiplicity of 74.1 (frames 1–200), whereas determinations from the mergings of crystals best to worst required nearly double that multiplicity (123.3 for crystals 1′ to 4′). We attribute this improvement to reduced radiation damage at lower wedged multiplicity. For DnaK, the substructure success rate peaks on merging of just five wedges (frames 1–250) and then falls off, which may also reflect the effects of radiation damage (Table 5 ▶ *b*). For HK9_S_, inclusion of the last two wedges even had a deleterious effect on substructure determination in that CC_weak_ from *SHELXD* decreased and map quality as judged by MapCC was nearly flat beyond wedge 4. These effects are also consistent with radiation damage that is mitigated by restricting exposure.

For the radiation doses used in these studies, we find that the quality of the electron-density maps (MapCC) improves asymptotically with wedged multiplicity (Fig. 10 ▶; see also Fig. 3 of Liu *et al.*, 2012 ▶), which implies that the damaged data contribute positively even if marginally. Nevertheless, there is a clear strategic advantage in limiting the radiation dose. This can be accomplished while still reaching adequate multiplicity for structure solution by combining measurements from many crystals with limited exposure for each. Since radiation damage is disordering as well as destructive, reducing high-angle diffraction first (Hendrickson, 1976 ▶), this limited-exposure approach is expected to improve resolution as well as the overall phasing statistics. In any case, it is evident from the results in Fig. 10 ▶ that wedge-by-wedge merging is advantageous, at least for analyzing the effects of radiation damage.

### 4.4. Identification of ionic constituents   

Many biologically relevant metal ions (Na^+^, Mg^2+^, K^+^ and Ca^2+^) and anions of interest (phosphate, sulfate and chloride) feature low-*Z* elements (*Z* ≤ 20). Although the chemical environments of such ions in crystal structures give important clues for identification, such sites can be ambiguous and especially so at low resolution. Anomalous scattering offers a means of direct identification, but the absorption edges for such light elements are far away from the X-ray energies commonly used in X-ray crystallography. Therefore, the direct identification of low-*Z* ions is challenging, especially for the lightest ions Na^+^ and Mg^2+^. Often, the indirect alternative of replacing a candidate ion with a chemical relative is used (Zhou & MacKinnon, 2003 ▶; Morth *et al.*, 2007 ▶; Kazantsev *et al.*, 2009 ▶). Examples include replacement of Na^+^ and K^+^ by Rb^+^ or Tl^+^; of Mg^2+^ and Ca^2+^ by Sr^2+^ or a lanthanide such as Yb^3+^; of Cl^−^ by Br^−^ or I^−^ and of SO_4_^2−^ by SeO_4_^2−^. Such replacements are never perfect, however, and direct *in situ* identification will certainly be advantageous. Others have used low-energy X-ray diffraction to show that diverse, and sometimes unexpected, ions are components of protein crystal structures (Mueller-Dieckmann *et al.*, 2007 ▶; Raaf *et al.*, 2008 ▶); however, previous studies have relied on chemical composition and environment for elemental identification.

The procedure that we have devised here for detecting and identifying low-*Z* ions directly from anomalous scattering signals is straightforward and robust. For site detection, we used Bijvoet-difference Fourier syntheses; for elemental identification, we used *f*′′ refinements against Bijvoet mates. The procedure worked effectively for all five native SAD structures in this study (Table 6 ▶ and Fig. 8 ▶; see also Fig. 2 of Liu *et al.*, 2012 ▶). Once an atomic structure is known, the same procedure could be used with a single-crystal data set, of course; however, multi-crystal averaging clearly improves the signals (Fig. 8 ▶). We used 7.1 keV X-rays in the current studies, whereas a lower X-ray energy could be beneficial owing to the increased *f*′′ values. It is worth noting that variable site occupancy is a potential complication for *f*′′ refinement since occupancy and scattering strength are redundant parameters. Occupancy is usually fixed at 1.0 for protein atoms unless alternate conformations are present; however, ions may be present at less than unit occupancy. For refinements at moderate resolution, *B* factors and occupancy parameters are highly correlated; however, ions or other ligands tend to have *B* factors that are similar to those of the protein atoms to which they are coordinated. Therefore, we fixed the *B* factors of ions to be at the average of those for associated protein atoms; we next refined occupancy parameters in a conventional refinement and finally we refined the *f*′′ anomalous scattering factors with occupancies fixed at these values. Notice that positional and *B*-factor parameters are mainly determined by the predominant normal scattering contributions; Bijvoet differences depend uniquely on *f*′′ values. The refined *f*′′ value provides an orthogonal check that must be consistent with the normal scattering identity used for atomic refinement.

### 4.5. Prospects for optimization of low-energy diffraction experiments   

We show in these studies that native SAD phasing can be effective even with X-rays at the modestly low energy of the Fe *K* edge and even with bending-magnet radiation. Experiments at lower energy promise stronger anomalous signals (Fig. 1 ▶ *a*), and with appropriate experimental designs the complications from absorption (Fig. 1 ▶ *b*) and incoherent scattering (Fig. 1 ▶ *c*) can be addressed very effectively. With the brightness of an undulator source, beam size and divergence can be reduced for better data quality. With noise-free readout over a wide dynamic range, pixel-array detectors can record diffraction data with improved accuracy (Mueller *et al.*, 2012 ▶). With improved methods for sample handling and automation, improved data sets composed from many dozens or hundreds of crystals can be envisioned. Moreover, with an advanced microdiffraction beamline, the smaller samples needed to take full advantage of lower X-ray energies for improved anomalous signals (Fig. 2 ▶). With lower X-ray energy, more challenging native SAD experiments, such as larger structures at lower resolution, might become routinely feasible. For energies below ∼4 keV (λ > ∼3 Å), flat detectors become problematic because of high scattering angles even for modestly fine Bragg spacings (Fig. 1 ▶ *d*) and detectors with quasi-spherical or quasi-cylindrical geometry are needed. Indeed, there is a strong case for synchrotron beamlines and instrumentation dedicated to low-energy diffraction experiments.

The data-processing procedures could also be optimized. For example, an improved local-scaling algorithm that takes better advantage of inverse-beam data could be used to reduce nonsystematic errors. A better weighting scheme could also be developed and used to enhance anomalous signals in merged data. Further optimization of crystal clustering and sorting procedures would be advantageous, especially when envisioning incomplete data sets from large numbers of microcrystals.

### 4.6. Prospects for routine use of native SAD structure determination   

We believe that procedures of the kind described here can have very broad applicability, and especially so with optimizations of the kind described above. We devised our multi-crystal native SAD procedures in the course of applications to five crystal structures (Table 2 ▶, Fig. 9 ▶) that are not atypical of the range of contemporary structural problems. They include structures with up to 1200 amino-acid residues in the asymmetric unit and have resolution limits as low as 2.8 Å. As of 10 April 2012, 90% of PDB entries from X-ray crystallography (63 559 out of 70 739) were determined at 2.8 Å resolution or higher. For *de novo* structures solved by SAD, the fraction at 2.8 Å resolution or better increases to 96% (5077 out of 5286). Thus, we suggest a broad reach for the method.

Structure determination by multi-crystal native SAD phasing is distinguished from current practice in requiring a supply of equivalent crystals, and meeting this condition may be an obstacle in some cases; however, an abundance of crystals is quite common and such production is likely to increase should the new technology take hold. At present, SeMet SAD is the predominant method for solving crystal structures *de novo* and molecular replacement dominates overall. With anticipated advances in synchrotron instrumentation and automation, it is plausible to expect that multi-crystal native SAD phasing could come to predominate for *de novo* structure determination, perhaps incorporating molecular replacements to aid in substructure determination. As an added benefit, such analyses could routinely include the discovery and reliable identification of associated ions such as Na^+^, Mg^2+^, K^+^ and Cl^−^, often troublesome heretofore.

## Figures and Tables

**Table 1:** *De novo* SAD PDB depositions PDB entries are as of 10 April 2012. *De novo* low-resolution SAD structures as defined here have *d*_min_ 3.5. *De novo* native SAD structures are defined as having no preceding PDB deposits and as not containing atoms heavier than atomic number 20. Thus, native SAD structures that include elements with *Z* > 20, such as Xe, I, Zn, Fe or Mn, are excluded. Structures with fewer than 40 amino-acid residues are also excluded. Depositions reported from the current studies (3va9, 3tx3, 3tbd, 3o1i and DnaK) are also excluded. PDB entries shown in bold have *d*_min_ > 2.0; all others are at higher resolution. Because entries in the PDB field ‘METHOD USED TO DETERMINE THE STRUCTURE’ are self-reported and are without uniform definition, it is possible that structures not listed here were also determined by native-SAD.

Year	All SAD	Low-resolution SAD	Only light-atom SAD	PDB codes of native only light-atom SAD structures
1981	1	0	1	1crn
				
1996	5	0	0	
1997	1	0	0	
1998	2	0	0	
1999	1	0	0	
2000	4	0	1	1el4
2001	23	0	0	
2002	52	0	2	1l7l 1o81
2003	160	1	3	**1p65** 1r7j 1rtt
2004	344	0	9	1tk1 1tov **1u8s** 1vka 1vkb 1vkq 1wf3 **1yav** 1ybz
2005	432	1	7	1ynb 1yoc 1z96 1zd0 **2azp** 2fbn 1zld
2006	520	2	9	**2dg82gnn** 2hly 2hq8 2hxp **2hzg2i522ja4** 2nxv
2007	686	6	10	2e6u **2qdn** 2qt7 2qvo 2rek 2v84 2yzq 2yzy **2zb92zcx**
2008	715	2	5	3c0f 3e19 3du1 **3e56** 3faj
2009	868	4	5	2wg7 2zy6 3g7n 3gb5 **3i0t**
2010	829	8	2	3faj 2xu8
2011	571	6	2	**3rqr** 2yil
2012	72	1	2	4ddj 4dlq
Total	5286	32	58	

**Table 2:** Summary of native SAD structure determinations

	HK9_S_	Netrin G2	CysZ	TorTTorS_S_	DnaK
No. of crystals	6	5	7	13	5
Space group	*I*4_1_22	*P*3_2_21	*C*2	*C*222_1_	*I*422
Resolution ()	2.3	2.3	2.3	2.8	2.3
Unique ordered residues	127	312	453	1148	1200
Unique S atoms	3	26	20	28	32
Other anomalous scatterers	Cl	Ca^2+^	Cl, SO_4_^2^	SO_4_^2^	SO_4_^2^, ATP, Mg^2+^
Total anomalous substructure	4	27	25	31	52
PDB entry	3va9	3tbd	3tx3	3o1i	4jn4

**Table 3:** Data-collection and reduction statistics Values in parentheses are for the highest resolution shell. The inverse-beam mode of data collection was used to collect all data sets except for data set 2. Inverse-beam mode is denoted by *N* + *N*, where *N* is the number of frames.

Crystal/data set	1	2	3	4	5	All
Unit-cell parameters						
*a* ()	292.270	291.284	291.969	291.595	291.578	291.739
*c* ()	99.551	99.501	99.576	99.441	99.428	99.500
No. of frames	400 + 400	400 + 400	400 + 400	400 + 400	400 + 400	4000
Bragg spacings ()	402.30 (2.362.30)	402.30 (2.362.30)	302.30 (2.362.30)	402.30 (2.362.30)	402.50 (2.572.50)	402.30 (2.362.30)
Measurements	2819882	2994574	2835994	2999571	2384284	14034502
Multiplicity	29.9 (11.6)	31.7 (29.8)	30.1 (15.9)	31.7 (30.6)	32.3 (31.2)	148.5 (87.1)
Completeness (%)	99.7 (96.3)	100.0 (100.0)	99.9 (99.3)	100.0 (100.0)	99.9 (98.9)	100.0 (100.0)
*R*_meas_ †	0.121 (0.630)	0.093 (0.388)	0.112 (0.493)	0.102 (0.554)	0.158 (0.690)	0.125 (0.498)
*R*_p.i.m._ ‡	0.030 (0.254)	0.024 (0.099)	0.028 (0.171)	0.025 (0.139)	0.038 (0.171)	0.014 (0.074)
*I*/(*I*)§	29.3 (4.3)	35.2 (11.9)	30.7 (6.6)	37.0 (9.5)	29.1 (8.1)	64.5 (16.2)
RACC (%) [rank¶]	67.3 [3]	70.3 [2]	63.7 [4]	74.2 [1]	52.6 [5]	100.0
*F*/(*F*)††	0.93	0.91	0.96	1.10	0.92	1.67
Anomalous CC‡‡ (%)	6.8	2.1	4.0	14.2	8.3	41.8

† *R*_meas_ is the redundancy-independent (multiplicity-weighted) *R*_merge_ as reported from *SCALA*.

‡ *R*_p.i.m._ is the precision-indicating (multiplicity-weighted) *R*_merge_ as reported from *SCALA*.

§ *I*/(*I*) = *I*(*hkl*)/[*I*(*hkl*)], where *I*(*hkl*) is the weighted mean of all measurements for reflection *hkl* and [*I*(*hkl*)] is the standard deviation of the weighted mean. The values are as reported from *SCALA* as Mn(I/sd).

¶ Rank of the anomalous correlation coefficient of individual data sets to the merged data.

†† *F*/(*F*) is the average anomalous signal from data truncated to *d*_min_ = 3.5. The values are derived using *CCP*4 programs and are computed by *SFTOOLS* as |*F*|/(*F*), where *F* = |*F*(*h*)| |*F*(*h*)|.

‡‡ Anomalous correlation coefficient evaluated from data truncated to *d*_min_ = 3.5.

**(a):** Phasing of RACC-ordered multi-crystal data sets.

Crystal/data set	1 to 1	1 to 2	1 to 3	1 to 4	1 to 5
Multiplicity	31.7 (30.6)	63.4 (60.3)	93.2 (71.4)	123.3 (87.1)	148.5 (87.1)
*I*/(*I*)	37.0 (9.5)	46.1 (14.6)	53.7 (15.1)	61.0 (16.1)	64.5 (16.2)
ACC (%)	14.2	17.2	29.6	37.2	41.8
Substructure success rate†	0	0	0	20	36
Maximum CC_all_ (%)	15.04	16.57	17.27	37.22	39.30
Maximum CC_weak_ (%)	4.16	4.49	5.57	22.42	23.84
FOM‡	0.231	0.254	0.266	0.279	0.309
Map CC before *DM* § (%)	36.0	41.7	43.8	45.3	46.6
Map CC after *DM* [Table-fn tfn10] (%)	73.0	76.1	86.1	83.8	85.3
No. of residues built[Table-fn tfn11]	1061/1200	1069/1200	1088/1200	1106/1200	1117/1200
Estimated model correctness[Table-fn tfn12] (%)	96.5	96.6	97.0	97.3	97.4

**(b):** Phasing of inversely reordered multi-crystal data sets.

Crystal/data set	5 to 5	5 to 4	5 to 3	5 to 2	5 to 1
Multiplicity	32.3 (31.2)	55.3 (15.8)	85.1 (27.0)	116.7 (56.6)	148.5 (87.1)
*I*/(*I*)	29.1 (8.1)	36.5 (6.6)	45.8 (7.7)	55.7 (13.4)	64.5 (16.2)
ACC (%)	8.3	14.9	27.1	34.3	41.8
Substructure success rate†	0	0	0	14	36
Maximum CC_all_ (%)	15.10	15.98	16.64	36.73	39.30
Maximum CC_weak_ (%)	4.26	4.79	5.61	21.33	23.84
FOM‡	0.191	0.204	0.231	0.267	0.309
Map CC before *DM* § (%)	24.4	36.9	40.5	44.1	46.6
Map CC after *DM* [Table-fn tfn10] (%)	44.9	77.4	78.1	79.0	85.3
No. of residues built[Table-fn tfn11]	481/1200	1078/1200	1104/1200	1108/1200	1117/1200
Estimated model correctness[Table-fn tfn12] (%)	31.4	97.0	97.3	97.4	97.4

† *SHELXD* solutions per 10000 attempts, resolution cutoff 3.8.

‡ Figure of merit.

§ Correlation coefficient between experimental (no density modification) and model-phased maps.

¶ Correlation coefficient between experimental (density-modified) and model-phased maps.

†† The number of residues built by *ARP*/*wARP* *versus* ordered residues in the refined model.

‡‡ Reported by *ARP*/*wARP*.

**(a):** Successive wedges.

Wedge (frames)	1 (150)	2 (51100)	3 (101150)	4 (151200)	5 (201250)	6 (251300)	7 (301350)	8 (351400)
Multiplicity	18.2 (10.9)	18.6 (10.8)	18.7 (10.9)	18.7 (10.9)	18.7 (11.0)	18.7 (11.1)	18.7 (11.2)	18.5 (11.3)
Completeness (%)	99.6 (97.1)	99.7 (98.8)	99.9 (99.4)	99.9 (99.8)	99.9 (99.5)	99.9 (99.8)	99.5 (97.8)	99.6 (98.2)
*I*/(*I*)	30.4 (7.7)	29.9 (7.4)	29.0 (6.8)	28.3 (6.6)	25.6 (5.4)	24.2 (4.9)	23.9 (4.1)	23.5 (3.9)
ACC (%)	3.9	4.5	1.6	0.6	0.2	0.5	4.6	0.4
MapCC before *DM* (%)	33.4	33.3	30.1	29.3	28.4	22.2	25.6	26.4
MapCC after *DM* (%)	65.1	68.0	53.5	51.5	50.0	39.1	42.9	44.8
No. of residues built	1079/1200	563/1200	519/1200	471/1200	502/1200	482/1200	515/1200	468/1200
Estimated model correctness (%)	96.9	63.3	58.6	46.5	50.6	49.9	50.6	50.3

**(b):** Accumulated wedges.

Wedge (frames)	1 (150)	2 (1100)	3 (1150)	4 (1200)	5 (1250)	6 (1300)	7 (1350)	8 (1400)
Multiplicity	18.2 (10.9)	36.7 (21.5)	55.4 (32.1)	74.1 (43.0)	92.8 (54.0)	111.5 (65.0)	130.1 (76.0)	148.5 (87.1)
Completeness (%)	99.6 (97.1)	99.9 (99.1)	100.0 (99.9)	100.0 (100.0)	100.0 (100.0)	100.0 (100.0)	100.0 (100.0)	100.0 (100.0)
*I*/(*I*)	30.4 (7.7)	40.6 (10.4)	49.0 (12.4)	54.0 (13.8)	57.7 (14.9)	60.8 (15.7)	63.9 (16.0)	64.5 (16.2)
ACC (%)	3.9	15.8	23.6	29.7	32.9	36.7	39.6	41.8
Substructure success rate	0	0	0	26	70	36	44	36
Maximum CC_all_ (%)				35.62	38.78	39.29	39.24	39.30
Maximum CC_weak_ (%)				21.28	23.76	23.84	24.40	23.84
FOM	0.212	0.262	0.282	0.300	0.296	0.300	0.300	0.309
MapCC before *DM* (%)	33.4	40.5	43.1	44.0	45.6	45.8	46.2	46.6
MapCC after *DM* (%)	65.1	75.7	75.3	81.3	84.6	85.0	85.2	85.3
No. of residues built	1079/1200	1108/1200	1107/1200	1112/1200	1098/1200	1109/1200	1109/1200	1117/1200
Estimated model correctness (%)	96.9	97.3	97.3	97.4	97.2	97.2	97.2	97.4

**Table 6:** Scattering-factor refinements for anomalously scattering elements

		*f* (refined)				
Atom (*Z*)	*f* (calc)	DnaK	HK9_S_	Netrin G2	CysZ	TorTTorS_S_
Ca (20)	1.598			1.501		
K (19)	1.329					
Cl (17)	0.883		0.801		0.797	
S_protein_ (16)	0.699	0.614	0.636	0.635	0.667	0.600
S_sulfate_ (16)	0.699	0.646			0.565	0.699
P (15)	0.550	0.491				
Mg (12)	0.227	0.250				
Na (11)	0.159					
